# The Sixth Annual Commencement of the Dental Department of the University of Maryland

**Published:** 1888-04

**Authors:** 


THE SIXTH ANNUAL COMMENCEMENT OF THE DENTAL DEPARTMENT OF THE UNIVERSITY OF MARYLAND was held at the Academy of Music, Baltimore, Md., on Wednesday, March 14th, 1888.

The address to the graduates was delivered by Professor Ferdinand J. S. Gorgas.

The number of matriculates for the session was one hundred and nine.

The degree of D. D. S. was conferred on the following graduates by Hon. S. Teackle Wallis, LL. D., provost of the university.

| | |
|---|---|
| Benjamin F. Baer, - - | Pennsylvania |
| Robert A. Bates, - - - | Virginia |
| John H. Bean, - - - | Massachusetts |
| William C. Berry, - - - | Virginia |
| Thomas C. Blackiston, - - | W. Virginia |
| George W. Blakeslee, - - | New York |
| Joseph H. Burgess, - - | S. Carolina |
| M. O. Burkholder, - - - | Virginia |
| Theodore A. Cross, - - | W. Virginia |
| Samuel S. Daniel, - - - | S. Carolina |
| L. Wilson Davis, - - - | Maryland |
| John W. Dean, - - - | W. Virginia |
| Wm. E. Dieffenderfer, M. D., - | D. Columbia |
| Manoog D. Dinjian, - - - | Turkey |
| George T. Feldmeyer, - - - | Maryland |
| J. Edgar Fitzgerald, - - | Maryland |
| Julian Gartrell, - - - - | Maryland |
| Clarence J. Grieves, - - | Maryland |
| John M. Hamlet, - - - | Virginia |
| Charles E. Harper, - - | Virginia |
| P. Edmond Hines, - - | N. Carolina |
| Charles R. Holt, - - - | New York |

| | |
|---|---|
| Charles P. Hubley, - - | Pennsylvania |
| Iræenus P. Jeter, - - - | S. Carolina |
| John A. Keepers, - - | Pennsylvania |
| Robert E. Lee, - - - | S. Carolina |
| Sylvester K. Marshall, - - | Maryland |
| A. D. McConachie, - - - | Canada |
| Thos. J. McLauchlin, - - | S. Carolina |
| Gerhard W. Muller, - - | Germany |
| Geo. F. Nelson, M. D., - - | Maryland |
| Frank H. Page, - - - | Canada |
| Francis E. Rambo, - - - | Georgia |
| Stafford Rambo, - - - | Georgia |
| Robert P. Rawlinson, - - | S. America |
| Harry J. Ray, - - - | S. Carolina |
| Edgar G. Smith, - - | New York |
| Edgar L. Smith, - - - | W. Virginia |
| Howard M. Smith, - - - | Virginia |
| James P. Smith, - - - | Virginia |
| Daniel B. Snively, - - | Pennsylvania |
| Frank Ryland Steel, - - - | Virginia |
| A. Zachary Taylor, - - | N. Carolina |
| Frederick P. Todd, - - - | Maryland |
| Joseph J. Vegas, - - | New York |
| Willis E. Watts, - - - | New York |
| Jacob L. Weirich, - - | Pennsylvania |
| Fred. M. Wheeler, - - | New Hampshire |
| Robert A. Wilbur, - - | S. Carolina |
| George L. Wilcox, - - - | New York |
| Frank M. Willis, - - | South Carolina |

